# Direct modeling of the crude probability of cancer death and the number of life years lost due to cancer without the need of cause of death: a pseudo-observation approach in the relative survival setting

**DOI:** 10.1093/biostatistics/kxaa017

**Published:** 2020-05-06

**Authors:** Dimitra-Kleio Kipourou, Maja Pohar Perme, Bernard Rachet, Aurelien Belot

**Affiliations:** 1 Cancer Survival Group, Faculty of Epidemiology and Population Health, Department of Non-Communicable Disease Epidemiology, London School of Hygiene & Tropical Medicine, London WC1E 7HT, UK; 2 Institute for Biostatistics and Medical Informatics, Faculty of Medicine, University of Ljubljana, Ljubljana, Slovenia

**Keywords:** Competing risks, Crude probability of death, Number of life years lost, Pseudo-observations, Relative survival

## Abstract

In population-based cancer studies, net survival is a crucial measure for population comparison purposes. However, alternative measures, namely the crude probability of death (CPr) and the number of life years lost (LYL) due to death according to different causes, are useful as complementary measures for reflecting different dimensions in terms of prognosis, treatment choice, or development of a control strategy. When the cause of death (COD) information is available, both measures can be estimated in competing risks setting using either cause-specific or subdistribution hazard regression models or with the pseudo-observation approach through direct modeling. We extended the pseudo-observation approach in order to model the CPr and the LYL due to different causes when information on COD is unavailable or unreliable (i.e., in relative survival setting). In a simulation study, we assessed the performance of the proposed approach in estimating regression parameters and examined models with different link functions that can provide an easier interpretation of the parameters. We showed that the pseudo-observation approach performs well for both measures and we illustrated their use on cervical cancer data from the England population-based cancer registry. A tutorial showing how to implement the method in R software is also provided.

## 1. Introduction

When aiming to describe the survival experience of a group of individuals, the estimation of the overall survival is usually of primary interest. However, when the goal is to describe the probabilities of dying from different causes, a further step is required in order to account for competing risks. Competing risks methods aim to identify covariates which not only affect the rate at which specific events occur but also the probability of occurrence of a specific event over time (Austin and Fine, 2017).

To perform a competing risks analysis with two events, say cancer death and death from other causes, we often rely on the cause of death (COD) information attributed to each individual, assuming that this is available and reliable. Two types of hazards, namely the cause-specific and the subdistribution hazard, may be used. Unlike cause-specific hazard, subdistribution hazard is useful for estimating covariate effects on the event-specific probability since it quantifies the effect of a covariate attributed to the direct effect of making the event more or less likely to occur or because of the indirect effect caused by the occurrence of other events (Dignam and Zhang, 2012). However, this leads to a complicated interpretation thus, working with cause-specific hazard might be preferred even though it does not directly describe the covariate effect on the probabilities (Andersen and Keiding, 2012).

Nevertheless, the use of routinely collected population-based registry data involves additional methodological challenges due to the absence of reliable information on individual COD, calling for methods defined within the *relative survival* setting (Pohar Perme *and others*, 2016). In this setting, the observed mortality hazard is split into two mortality hazards: the expected or population mortality hazard (assumed known and provided by population life tables) and the excess mortality hazard, which is the main quantity of interest. The excess mortality hazard in the relative survival setting is the equivalent of cause-specific (here cancer-specific) hazard in classical competing risks setting. The most frequently used indicator derived from the excess mortality hazard is *net survival* (Pohar Perme *and others*, 2012), which describes the probability of surviving when assuming that the cancer under study is the only possible COD. Net survival is of interest when making comparisons between populations since it is independent of the competing risks of death, which may differ between these populations (Allemani *and others*, 2018; De Angelis *and others*, 2014).

Despite the usefulness of net survival, communicating survival statistics is complicated and must involve various indicators, as to reflect different dimensions in terms of prognosis, treatment choice, or development of a control strategy. Towards this direction alternative indicators like (i) the *Crude Probability of Death* (CPr) from a given cause (Cronin and Feuer, 2000; Mariotto *and others*, 2014; Pfeiffer and Gail, 2017), also called cause-specific cumulative incidence function, and (ii) the number of *Life Years Lost* (LYL) due to a given cause (Andersen, 2013), can be used as complementary tools in order to provide a multi-perspective approach (Belot *and others*, 2019).

Crude probabilities can be estimated nonparametrically using the Aalen–Johansen estimator (Satagopan *and others*, 2004; Geskus, 2015), or modeled in cause-specific setting with regression models on the cause-specific hazards (Pfeiffer and Gail, 2017; Kipourou *and others*, 2019) or on the subdistribution hazards (Fine and Gray, 1999; Geskus, 2015; Mozumder *and others*, 2018) or modeled in relative survival setting using regression models on the excess hazard (Lambert *and others*, 2010; Eloranta *and others*, 2013; Charvat *and others*, 2013; Charvat *and others*, 2016). In cause-specific setting, the pseudo-observation approach is another option (Andersen *and others*, 2003; Klein and Andersen, 2005; Andersen and Pohar Perme, 2010) allowing for the direct modeling of probabilities. In relative survival setting, CPr can be estimated nonparametrically or indirectly via regression modeling of the excess hazard but not through direct modeling. Similarly, although estimation and modeling of LYL can be implemented in cause-specific setting (Andersen, 2013), modeling them in relative survival setting has yet to be implemented.

The scope of this article is to present a way of modeling directly the CPr and LYL due to the disease of interest and other causes in relative survival setting (i.e., when COD is not available) according to some covariates of interest. We chose to extend the most general method, i.e., the pseudo-observation method (Andersen *and others*, 2003; Klein and Andersen, 2005; Andersen and Pohar Perme, 2010; Andersen, 2013), which can be applied to both measures. The main idea is based on the fact that when there is censoring we do not always observe the random variable (e.g., time to event). By generating pseudo-observations at specific time points, we replace the whole set of incompletely observed random variables with a complete set of their pseudo-observations. These are later modeled with standard methods like generalized linear models (GLM) or generalized estimating equations (GEE) in order to quantify covariate effects directly on the indicators of interest.

The remainder of the article is organized as follows: Section 2 provides a general description of the pseudo-observation approach and details how this can be adapted in relative survival setting in order to model directly the CPr and LYL. In Section 3, we assessed the performance of the method in its ability to estimate the regression parameters of interest and examined models with different link functions using simulations. In Section 4, we applied the new method on population-based cancer registry data of women diagnosed with cervical cancer in England between 2008 and 2010, and discussed the useful interpretation that can be gained from these models. Lastly, Section 5 summarizes the results and presents ideas for further research.

## 2. Methods

### 2.1. Pseudo-observations

The method based on pseudo-observations provides a general framework that enables the direct modeling of a given statistical measure (e.g., survival probability) as a function of some covariates of interest. Pseudo-observations (also called pseudo-values) were first described for multistate models (Andersen *and others*, 2003), and since then many extensions were proposed [e.g., for cause-specific cumulative probabilities within the classical competing risks setting (Klein and Andersen, 2005; Moreno-Betancur and Latouche, 2013) or for the restricted mean survival time (Andersen *and others*, 2004)]. This approach requires the existence of an (approximately) unbiased estimator of the measure of interest (Andersen and Pohar Perme, 2010). While its usefulness goes beyond modeling [as it can be extended to providing goodness-of-fit methods (Andersen and Pohar Perme, 2010; Pavlič *and others*, 2018)], we focus on the modeling part here, and summarize the main steps for their use when analyzing time to event data.

For an individual $i = 1,\ldots,n$, let $Y_i$ be independent and identically distributed random variables (e.g., time since diagnosis up to death), and $\boldsymbol{X}_i$ a $p$-dimensional vector of (time-fixed) covariates. As it is often the case with time to event data analysis, $Y_i$ is not always observed due to censoring. Pseudo-observations are useful when information on $Y_i$ is not available, and our interest lies on modeling the $E\left[f(Y_i)|\boldsymbol{X}_i\right]$ for a given function $f$.

The main idea of pseudo-observations relies on the fact that even with incomplete (censored) data we can still derive the marginal expectation $E\left[f(Y)\right]$. Assuming that a consistent and (approximately) unbiased estimator $\hat\theta$ exists for $\theta=E\left[f(Y)\right]$ [e.g., the Kaplan–Meier estimator for the survival probability, or the Aalen–Johansen estimator for the cause-specific cumulative incidence function (Geskus, 2015)], then the possibly unknown $f(Y_i)$ could be replaced by its pseudo-observation (Andersen and Pohar Perme, 2010).

Pseudo-observations are computed for every individual regardless of the availability of the $f(Y_i)$ at specific times. Thus, the pseudo-observation for $f(Y_i)$ is defined for individual $i=1,\ldots,n$ at a given time $t$ as

(2.1) \begin{equation*} \tilde\theta_{i}=n\cdot\hat{\theta}-(n-1)\cdot\hat{\theta}^{-i}, \label{leave-one-out-estimator} \end{equation*}

where $\hat{\theta}$ is the estimator at time $t$ based on the whole sample and $\hat{\theta}^{-i}$ is the estimator at time $t$ based on the sample of size $(n-1)$, obtained by eliminating individual $i$ from the whole sample. Intuitively, the pseudo-observation $\tilde\theta_{i}$ can be seen as the “contribution” of the individual $i$ to the $E\left[f(Y_{i})|\boldsymbol{X}_{i}\right]$, estimated on the basis of the full sample at time $t$ (Andersen and Pohar Perme, 2010).

Pseudo-observations may be calculated at several time points. In this case, the pseudo-observation $\tilde{\boldsymbol{\theta}}_i$ is $m$-dimensional (i.e., $(\tilde{\boldsymbol{\theta}}_i)_j=\tilde\theta_{ij}, \quad j=1,\ldots,m$) and represents the vector $f(\boldsymbol{Y}_{i})$ ($\boldsymbol{Y}_{i}=(Y_{i1},\cdots,Y_{im}))$ with entries $f(Y_{ij})$. These pseudo-observations may be used as the outcome variables in a generalized linear regression model in order to derive the covariate effects on the outcome of interest as

(2.2) \begin{equation*} g\{E[f(Y_{ij})|\boldsymbol{X}_{i}]\}={\boldsymbol{\alpha}}_j+{\boldsymbol{\gamma}}^\top\boldsymbol{X}_{i}=\boldsymbol{\beta}^\top\boldsymbol{X}_{ij}^*, \qquad i=1,\ldots,n \quad j=1,\ldots,m, \label{glm2} \end{equation*}

where $g$ is a monotone differentiable link function and $\boldsymbol{X}_{ij}^*$ is a ($m+p$ dimensional) vector including the indicators of the time points and the covariates $\boldsymbol{X}_i$, $\boldsymbol{X}_{ij}^*=(e_j^\top, \boldsymbol{X}_i^\top)^\top$, where $e_j$ is the m-dimensional vector with 1 on the $j$th entry and 0 otherwise (Andersen and Pohar Perme, 2010). Adding interaction terms (between covariates and time terms) would make $\boldsymbol{X}_{ij}^*$ higher dimensional.

Because the pseudo-observations for a given subject could not be considered as independent random variables, estimating the ($m+p$) regression parameters $\boldsymbol{\beta}$ is based on the GEE method (Liang and Zeger, 1986). The estimating equations to be solved are

(2.3) \begin{equation*} \sum_{i=1}^n \left(\frac{\partial}{\partial \boldsymbol{\beta}} g^{-1}(\boldsymbol{\beta}^\top\boldsymbol{X}_{i}^*)\right)^\top V_i^{-1} \left\{\tilde{\boldsymbol{\theta}}_i - g^{-1}(\boldsymbol{\beta}^\top\boldsymbol{X}_{i}^*) \right\}=0, \label{liangzeger} \end{equation*}

where $g^{-1}(\boldsymbol{\beta}^\top\boldsymbol{X}_{i}^*)$ is an m-dimensional vector ($g^{-1}(\boldsymbol{\beta}^\top\boldsymbol{X}_{i1}^*), \cdots, g^{-1}(\boldsymbol{\beta}^\top\boldsymbol{X}_{im}^*)$) and $V_i$ is a working covariance matrix with a pre-defined structure.

In order for the pseudo-observation approach to work, it has been shown that censoring should not depend on covariates (Graw *and others*, 2009), alternatively modified pseudo-observations should be applied (Binder *and others*, 2014). For the variance of the estimated regression parameters $\hat{\boldsymbol{\beta}}$, a sandwich estimator could be used (Andersen *and others*, 2003). Even if it has been shown that this might lead to inconsistent and upward biased results (especially in the case of large samples), this has an insignificant impact in practical applications (Jacobsen and Martinussen, 2016; Overgaard *and others*, 2017, 2018).

The user has various choices with respect to the link function $g$ and the structure of the working covariance matrix $V$. A clever choice of the latter may increase efficiency (Andersen, 2013), but we do not explore this further in this study.

### 2.2. The relative survival setting and the excess mortality hazard approach

The relative survival setting is a specific competing risks setting where, although the COD information is either missing or is unreliable, inference about the event/disease of interest can still be drawn under specific assumptions and conditions (detailed below). In this article, the disease of interest is a specific cancer and the time scale used for measuring the time to event is the time since cancer diagnosis.

In relative survival setting, we consider two sets of data: (i) data on time to death (but without COD information) from a cohort of patients with the specific cancer of interest and (ii) life tables of the general population in which all-cause hazard functions (stratified by some sociodemographic variables $\mathbf{z}$) are available (Pohar Perme *and others*, 2012). The main assumption we make here is that for an individual $i$, the observed hazard $\lambda_C(t;\boldsymbol{X}_i)$ described by the covariates $\boldsymbol{X}_i$ can be decomposed as the sum of the cancer-specific mortality hazard $\lambda_C(t;\boldsymbol{X}_i)$ and the hazard related to other causes $\lambda_P(t;\mathbf{z}_i)$ (with $\mathbf{z}_i \subset \boldsymbol{X}_i$):

(2.4) \begin{equation*} \lambda_O(t;\boldsymbol{X}_i) = \lambda_C(t;\boldsymbol{X}_i) + \lambda_P(t;\mathbf{z}_i). \end{equation*}

We further assume that $\lambda_P$ is equal to all-cause hazard of the general population within levels of $\mathbf{z}$. For this assumption to hold, the following conditions must be met:

- the specific cancer of interest is considered a negligible COD in the general population (Ederer, 1961). This is especially true when prevalence is low (i.e., rare cancers and younger age groups), but it might be unreasonable when focusing for example on older people with common cancers (e.g., prostate cancer) or when all cancer sites are combined (Hinchliffe *and others*, 2012; Talbäck and Dickman, 2011).
- the other-cause hazard of the general population is equal to the other-cause hazard of the study population within levels of $\mathbf{z}$. Moreover, within levels of $\mathbf{z}$, the other-cause hazard does not further depend on $\boldsymbol{X}$ nor on any (unmeasured) covariates. This latter condition may not be realistic for some cancers, and an adaptation of the method might be needed (Rubio *and others*, 2019).

In most situations, the minimum set of sociodemographic covariates $\mathbf{z}$ stratifying life tables (and therefore $\lambda_P$) is sex, age (in 1-year age-group), calendar year and geographical level. In some countries, additional stratifying variables may be available, such as deprivation level or ethnicity.

A discussion of the assumptions and related conditions that should be met for the relative survival setting to be valid can be found in Pavlič and Pohar Perme (2018).

### 2.3. Measures of interest in the relative survival setting

#### 2.3.1. CPr from a specific cause.

In the classical competing risks setting where the COD is available, the (cause $k$)-specific probability of death $F_k(t)$ (also called cumulative incidence function) represents the probability of dying from cause-$k$ before or at time $t$, and can be expressed as $F_k(t)=\int_0^tS(u-)\mathrm{d}\Lambda_k(u)$, where $S$ is the all-cause survival function and $\Lambda_k$ is the cumulative (cause $k$)-specific hazard.

In the relative survival framework, the CPr from cancer $F_\text{C}(t)$ is expressed as $F_\text{C}(t)=\int_{0}^{t} S(u-) \mathrm{d} \Lambda_C(u)$ (Cronin and Feuer, 2000; Lambert *and others*, 2010; Charvat *and others*, 2013). It may be estimated using the marginal cancer-specific hazard $\lambda_{\textit{C}}(t)$, this latter being defined as the combination of the *individual* cancer-specific hazards, $\lambda_{\textit{C}}(t,\boldsymbol{X})$ [see equations (5) and (6) in (Pohar Perme *and others*, 2012), while more details can be found in (Belot *and others*, 2019; Pohar Perme and Pavlic, 2018)]. Thus, it holds that

(2.5) \begin{equation*} \hat F_\text{C}(t)=\int_{0}^{t} \hat S(u-) \mathrm{d}\hat\Lambda_\textit{C}(u), \label{cif_rsC} \end{equation*}

where $\hat S(u-)$ is the Kaplan–Meier estimator of the overall survival and the estimator of the cancer-specific cumulative hazard is calculated as

$$\begin{equation*}
\mathrm{d}{\hat\Lambda_{\textit{C}}}(t)= \frac{\mathrm{d} N(t) - \displaystyle \sum_{i=1}^{n} Y_i(
t)\mathrm{d}\Lambda_{\textit{P}}(t,\mathbf{z}_i)}{Y(t)}.
\label{ExcessHaz}
\end{equation*}$$

Similarly, the CPr from other causes can be estimated as

(2.6) \begin{equation*} \hat F_\text{P}(t)=\int_{0}^{t} \hat S(u-) \mathrm{d} \hat \Lambda_\textit{P}(u), \label{cif_rsP} \end{equation*}

where

$$\begin{equation*}
\mathrm{d}{\hat\Lambda_{\textit{P}}}(t)= \frac{\displaystyle \sum_{i=1}^{n} Y_i(
t)\mathrm{d}\Lambda_{\textit{P}}(t,\mathbf{z}_i)}{Y(t)}
\label{PopHaz}
\end{equation*}$$

In both formulae, $\mathrm{d}\Lambda_{\textit{P}}$ is obtained through $\lambda_{\textit{P}}$, which is the population mortality hazard that an individual $i$ with covariates $\mathbf{z}_i$, $i=1, \ldots, n$, is exposed to at time $t$. $N(t)$ and $Y(t)$ are counting processes, where $N(t)$ is the number of individuals who have experienced an event of any type in $[0,t]$, and $Y(t)$ is the number of individuals who are still at risk at time $t$, obtained as the sum of indicators whether a person is still at risk, $Y(t)=\sum Y_i(t)$ (Klein and Andersen, 2005; Andersen and Pohar Perme, 2010; Pohar Perme and Pavlic, 2018).

This method of estimation is already provided in the R-package relsurv (Pohar Perme, 2018).

#### 2.3.2. Number of LYL due to a specific cause.

The expected LYL due to a specific cause (for a given time window) is a useful complementary indicator (Andersen, 2013), allowing for an easier interpretation of the results, which is expressed with time units. In clinical setting this indicator provides an interesting insight on prognosis, treatment choice, or the development of a control strategy.

Without distinguishing death from different causes, the LYL before time $\tau$ [compared to an immortal cohort (Andersen, 2013), i.e., where nobody dies before time $\tau$], may be expressed as

$$\begin{equation*}
L(0,\tau)=\tau-\int_{0}^{\tau}S(u)\mathrm{d} u.
\end{equation*}$$

The total LYL can be further decomposed according to COD in the classical competing risks setting as $L_k(0,\tau)=\int_0^\tau F_k(u)\mathrm{d} u,$ where $F_k(t)$ is the cause $k$-specific cumulative probability of death (Andersen, 2013). Therefore, following the same analogy as before, this decomposition can be extended to relative survival setting for the LYL due to cancer $L_{\textit{C}}$ and due to other causes $L_{\textit{P}}$ (Belot *and others*, 2019):

(2.7) \begin{equation*} L_{\textit{C}}(0,\tau)=\int_0^\tau F_\textit{C}(u)\mathrm{d} u, \quad L_{\textit{P}}(0,\tau)=\int_0^\tau F_\textit{P}(u)\mathrm{d} u. \label{lyl_rs1} \end{equation*}

Finally, by plugging into equation (2.7) the estimators (2.5) and (2.6) we can estimate the $\hat{L}_{\textit{C}}(0,\tau)$ and $\hat{L}_{\textit{P}}(0,\tau)$, respectively.

### 2.4. Pseudo-observations in the relative survival setting for estimating covariates effects on the CPr and the LYL due to different causes

The pseudo-observation for the CPr due to cancer for an individual $i$ at time $t$, $\tilde{F}_{\textit{C},it}$ is calculated [based on the equations (2.1) and (2.5)] as

(2.8) \begin{equation*} \tilde{F}_{\textit{C},it}=n\cdot\hat{F}_{\textit{C}}(t)-(n-1)\cdot\hat{F}_{\textit{C}}^{-i}(t). \end{equation*}

This pseudo-observation is defined at $m$ timepoints with $m$ varying between 5 and 10 different timepoints, which can be either equally spread or chosen based on quantiles of the overall survival time distribution (Klein and Andersen, 2005). The pseudo-observations for the CPr of death due to other causes are defined in the same way.

For the LYL due to cancer $L_{\textit{C}, i}(0,\tau)$ (respectively other cause, $L_{\textit{P}, i}(0,\tau)$ ), we compute only $m$=1 pseudo-observation at time $\tau$ for each individual [based on the equations (2.1), (2.7)] as

(2.9) \begin{equation*} \tilde{L}_{\textit{C}, i}(0,\tau)=n\cdot\hat{L}_{\textit{C}}(0,\tau)-(n-1)\cdot\hat{L}_{\textit{C}}(0,\tau)^{-i}. \end{equation*}

For both indicators, after calculating these pseudo-observations we generate a new data set in which every individual is assigned with $m$ pseudo-observations (corresponding to the $m$ time-points), which later will be used as the main outcome in a regression model (Andersen *and others*, 2003). A GEE model of the form $g(E[Y|\boldsymbol{X}_i])=\boldsymbol{\beta}^\top\boldsymbol{X}_{ij}^*$ is typically used, where $g$ is a link function, $\mathbf{\boldsymbol{\beta}}$ is the corresponding vector of $m+p$ regression parameters, and $\boldsymbol{X}_{ij}^*$ is a vector including the covariates for the individual $i$ ($\boldsymbol{X}_i$) as well as the intercept and the indicator functions of the ($m-1$) remaining timepoints.

#### 2.4.1. User choices: link function and working covariance matrix.

Interpretation of regression coefficients varies according to the link function used. For the CPr, most common $g$ link functions are the *cloglog*, *log*, and *identity*.

A *cloglog* link function on $F_\textit{C}(t)$ defined as $\log(-\log(1-F_\textit{C}))$ leads to similar regression coefficient estimates to those obtained with Fine and Gray model (Fine and Gray, 1999). In this case, the $\exp(\boldsymbol{\beta})$ is a hazard ratio which is related to the subdistribution hazard, i.e., the instantaneous rate of failure per time unit from cause $j$ among those who are either alive or have had a competing event at time $t$. Due to the complicated nature of this type of hazard ratios, the regression coefficients are interpreted in a qualitative (higher or lower than 1) rather than quantitative way (Andersen *and others*, 2012). Nonetheless, a test of statistical significance of a subdistribution hazard ratio provides a test of the covariate effect on the CPr (Austin and Fine, 2017).

A *log* link function provides regression coefficients with less complicated interpretation. The $\exp(\boldsymbol{\beta})$ obtained from a model with log link function gives an estimate of the relative risks (Overgaard *and others*, 2015) allowing for quantitative interpretations. However, constraining probabilities between [0,1] might be problematic in situations with high absolute risks or when extrapolating outside the data range (Lambert *and others*, 2017).

Additionally, an *identity* link function can be applied to CPr leading to regression coefficients that are interpreted as risk differences (Klein, 2006; Hansen *and others*, 2014). The identity link function is usually the link function of choice for models on LYL as well. In this case, the interpretation shows the additional life years that are lost due to a given cause. In both cases though, results might go beyond the admissible range which is set for each indicator and thus, one must be careful of predictions outside the observed limits.

The *logit* link function (not explored here) would be another option giving also convenient interpretations, i.e., odds ratios. This choice also suffers from certain drawbacks such as numerical instabilities for small values of time $t$ (Gerds *and others*, 2012).

We account for the correlation in the pseudo-observation data through the use of a specific structure of the working covariance matrix (Pekár and Brabec, 2018). The choice of a covariance matrix structure might vary between independence, exchangeable, autoregressive, and unstructured, although it is suggested that even the independence working covariance matrix is adequate (Klein and Andersen, 2005).

## 3. Simulation study

In this study we conjecture that the pseudo-observation approach for the relative survival setting will work in a similar way as in the classical competing risks setting and GEE would be a reasonable approach to yield both regression parameter and variance estimates. With a simulation study, we examine the validity of the method in practice. Simulations were performed in order to evaluate the frequentist properties of the proposed method based on pseudo-observations in its ability to estimate regression parameters of covariates associated to CPr and LYL due to death from cancer and from other causes.

### 3.1. Data generation and simulation design

We simulated $n_{\text{sim}}=500$ data sets with sample size of $N=\{300,1000\}$. Each individual was assigned with a vector of three covariates which includes information about sex, year of diagnosis, and age at diagnosis. Sex was simulated as binary drawn from a Bernoulli distribution with probability 0.5. Year of diagnosis was simulated as a continuous variable and sampled from a uniform distribution ranging from 2000 to 2003. Age at diagnosis was simulated as a continuous variable by first selecting an age class according to predefined probabilities [0.25 for age class [30,65), 0.35 for age class [65,75) and 0.4 for age class [75,80)] and then sampling from a class-specific uniform distribution (Belot *and others*, 2010).

This scenario tried to mimic what could be observed in real situations for colon cancer patients. We used a Generalized Weibull distribution with parameters ($\kappa, \rho, \alpha$) to model the subdistribution hazard (SDH). For individual $i$, the SDH related to cancer $\gamma_C$ was defined as

$$\gamma_C(t,\textit{Age}_i,\textit{Sex}_i,\textit{Year}_{i} )=\gamma_0(t)\exp\left\{\beta_{\textit{Age}}\textit{Age}_i+ \beta_{\textit{Sex}}\textit{Sex}_i+ \beta_{\textit{Year}}\textit{Year}_i\right\}\!,$$

where

$$\gamma_0(t)=\frac{\kappa\rho^\kappa t^{\kappa-1}}{1+\frac{(\rho t)^\kappa}{\alpha}}.$$

The parameters used for the baseline hazard, namely $\{\kappa, \rho, \alpha\}$, were set to $\{2, 1.6, 0.05\}$. The values used for the covariate regression parameters were $\beta_\textit{Age}=0.2$ (for 1 year increase), $\beta_{\textit{Sex}}=0.3$, and $\beta_{\textit{Year}}=0$, accounting for different strength in effect sizes; a very strong effect (age), a weak effect (sex), and a null effect (year). In this way, simulations include the most common covariates used in relative survival analyses.

We obtained the expected mortality $\lambda_{\textit{P}}$ from UK life tables based on some demographic characteristics, namely year, age, and sex (Danieli *and others*, 2012). The $\lambda_{\textit{P}}$ changed annually for a given age and sex and remained constant during a year hence, following a piecewise exponential distribution.

Using $\gamma_C$ and $\lambda_{\textit{P}}$, we obtained the cancer-specific hazard $\lambda_{\textit{C}}$ by adapting the approach described in Haller and Ulm (2014). The individual survival time (from any cause) was obtained using the inverse probability transform method (Bender *and others*, 2005; Belot *and others*, 2010). More information on the simulation algorithm is provided in Section 1 of Supplementary Material available at *Biostatistics* online.

We set the administrative censoring time ($C$) at 10 years and allowed for a separate distribution to account for drop outs, which followed an exponential distribution ($\lambda_\textit{d}=0.035$). This results in approximately 8% loss to follow-up, while the total amount of censoring in each data set was on average around 42%. A vital status indicator $\delta$ was created, $\delta=0$ for individual censored at $T$ and $\delta=1$ for those being dead at time $T$ (irrespective of COD).

### 3.2. Analysis of simulated data

For CPr from cancer and other causes, we tested three GEE models for the pseudo-observations: (a) a model with *log* link function, (b) a model with *cloglog* link function, and (c) a model with *identity* link function. All models were assuming independence working correlation and included the explanatory variables age at diagnosis, sex, and year of diagnosis.

To model LYL within 10 years from death caused by cancer or other causes, we fitted a GEE model with *identity* link function, explanatory variables: age at diagnosis, sex, and year of diagnosis, and independence covariance structure.

We calculated the following performance measures:

(1) bias, defined as the difference between the average of the $n_{\textrm{sim}}=500$ estimated values and the true value $\beta^*_0$: $\frac{1}{n_{\textrm{sim}}}\sum_{i=1}^{n_{\textrm{sim}}}\hat{\beta}_i-\beta^*_0$,
(2) empirical standard error $\sqrt{\frac{1}{n_\textrm{sim}-1}\sum_{i=1}^{n_\textrm{sim}}(\hat{\beta}_i- \bar \beta)^2}$, where $\bar \beta=\frac{1}{n_{\textrm{sim}}}\sum_{i=1}^{n_\textrm{sim}}\hat{\beta}_i$,
(3) model standard error $\sqrt{\frac{1}{n_{\textrm{sim}}}\sum_{i=1}^{n_\textrm{sim}}[\hat{{\textrm{Var}}}(\hat{\beta_i})]}$,
(4) root mean squared error $\sqrt{\frac{1}{n_{\textrm{sim}}}\sum_{i=1}^{n_{\textrm{sim}}}(\hat{\beta}_i-\beta^*_0)^2}$, and
(5) the coverage which is the proportion of samples in which the 95% confidence interval included $\beta^*_0$.

Using a cancer-specific subdistribution hazard model allows us to directly assess only the cancer-specific estimates provided by the model with the cloglog link function. For the other two link functions and the other causes with cloglog (where real population hazards were taken), the performance was assessed indirectly with the least false parameters (LFP) (Hjort, 1992; Beyersmann *and others*, 2009). The LFP were obtained after applying the same models described previously to a data set of 100 000 individuals, which was generated using the same simulation algorithm but without considering any drop outs (for more details please see Section 2 of Supplementary Material available at *Biostatistics* online). Both true and LFP were available for the cloglog, so by comparing those two we were able to evaluate the sensibility of the LFP as proxies of the true values. The LFP for model (b) for the cancer case were $(0.199, 0.299, 0.005)$ whereas the true (simulated) were $(0.2, 0.3, 0)$, validating this way of comparison.

Our computations were performed in R 3.2.0. We used the nonparametric method for the CPr provided by the R-package relsurv [version 2.1.1, function cmp.rel (Pohar Perme, 2018)], while GEE models were fitted with the R-package geepack (version 3.2.5, function geese).

### 3.3. Simulation results

#### 3.3.1. CPr of death from colon cancer and other causes.

Results shown in Table 1 suggested that regardless of the link function used, the regression parameter estimates of the covariate effects were almost unbiased with most of the coverage probabilities lying within the acceptable coverage range ($[0.931,0.969]$) for all parameter estimates and for any cause (cancer or other causes). Results were similar for both sample sizes although, for model (c) results seem to be slightly better when $N=1000$ due to a smaller bias in the larger sample size. In general, standard error was found to be adequately estimated with the models based on how close the empirical standard errors compared to model standard errors are. RMSEs were also reasonably low proving also good model performance.

**Table 1.** Simulation results: performance measures of regression parameter estimated using pseudo-observation and three models for the crude probabilities of death from cancer and from other causes; model (a) assumed a *log* link function, model (b) assumed a *cloglog* link function, and model (c) assumed an *identity* link function. In all models, the independence working covariance structure was used. The explanatory variables in all models were age at diagnosis, sex, and year of diagnosis. Results based on 500 simulated data sets with different sample sizes ($N=300,1000$).

Model	Cause	Covariate	LFP	$\hat \beta$		Bias ($\times 1000$)		empSE		ModSE		RMSE		Coverage$^\ddagger$	
				$N=300$	$N=1000$	$N=300$	$N=1000$	$N=300$	$N=1000$	$N=300$	$N=1000$	$N=300$	$N=1000$	$N=300$	$N=1000$
(a)	Cancer	Age	0.163	0.174	0.165	10.649	1.869	0.08	0.038	0.075	0.04	0.081	0.039	0.932	0.956
		Sex	0.24	0.263	0.249	22.386	8.587	0.218	0.118	0.21	0.115	0.219	0.118	0.948	0.944
		Year	0.004	−0.006	0	−10.162	−3.997	0.109	0.056	0.102	0.055	0.109	0.056	0.936	0.946
	Other causes	Age	0.693	0.709	0.694	16.23	1.664	0.105	0.056	0.111	0.058	0.107	0.056	0.952	0.944
		Sex	0.158	0.163	0.148	4.38	−10.76	0.164	0.079	0.158	0.083	0.164	0.08	0.932	0.966
		Year	−0.016	−0.012	−0.016	3.887	0.341	0.093	0.049	0.088	0.047	0.093	0.049	0.936	0.936
(b)	Cancer	Age	0.2†	0.212	0.202	12.071	1.623	0.098	0.047	0.09	0.048	0.098	0.047	0.932	0.95
		Sex	0.3†	0.325	0.309	25.02	8.801	0.261	0.143	0.253	0.139	0.262	0.143	0.948	0.948
		Year	0†	−0.007	0	−7.334	−0.037	0.137	0.07	0.13	0.07	0.137	0.07	0.936	0.94
	Other causes	Age	0.793	0.81	0.794	17.059	1.073	0.125	0.066	0.129	0.068	0.126	0.066	0.948	0.942
		Sex	0.194	0.195	0.184	1.128	−10.17	0.181	0.087	0.175	0.092	0.181	0.088	0.936	0.966
		Year	−0.019	−0.015	−0.019	4.625	0.758	0.104	0.055	0.098	0.052	0.104	0.055	0.938	0.938
(c)	Cancer	Age	0.04	0.04	0.04	−0.326	−0.259	0.015	0.008	0.015	0.008	0.015	0.008	0.926	0.946
		Sex	0.064	0.066	0.064	2.358	0.682	0.048	0.028	0.049	0.027	0.048	0.028	0.948	0.942
		Year	0.002	0	0	−2.068	−1.637	0.029	0.015	0.028	0.015	0.029	0.015	0.924	0.958
	Other causes	Age	0.037	0.037	0.038	−0.302	0.789	0.003	0.002	0.003	0.002	0.003	0.002	0.97	0.964
		Sex	0.019	0.022	0.019	3.329	0.795	0.009	0.005	0.009	0.005	0.009	0.005	0.932	0.962
		Year	−0.002	0.001	−0.003	2.718	−0.835	0.006	0.003	0.006	0.003	0.006	0.003	0.912	0.928

LFP, least false parameter; empSE, empirical standard error; ModSE, model standard error; RMSE, root mean squared error. $^\dagger$ True values $\ddagger$ Acceptable coverage range is calculated as $0.95\pm z_\alpha\sqrt{\frac{0.95\cdot0.05}{500}}=[0.931,0.969]$

The only exception to that is the regression parameter estimates in model (c) in the case of age (for cancer) and year (for both causes) when $N=300$. In all cases, standard error seemed to be well estimated thus, indicating that the bias in the estimator should be probably the reason for the problematic coverage probability. A different choice of working correlation structure would change both the regression parameter estimate and its variance, leading to a possibly better coverage probability, while model misspecification might be an additional issue which may be considered.

#### 3.3.2. Life years lost.

The regression parameters were well estimated when modeling the number of LYL due to each cause, with a very small bias and a good coverage (see Table 2). Only exception to that was the estimated regression parameter for the effect of sex and year in the case of other causes when $N=1000$. An overestimation of the standard error by the model might have inflated the coverage probability in case of sex, while bias seems to be the source of problem in the case of year. Another specification of the model including a change of the working covariance matrix would be additional things to consider.

**Table 2.** Simulation results: performance measures of regression parameter estimated using pseudo-observation and a model with *identity* link function and an independence working covariance matrix for the number of LYL due to cancer and due to other causes. The explanatory variables were age at diagnosis, sex, and year of diagnosis. Results based on 500 simulated data sets with different sample sizes (N=300,1000).

Cause	Covariate	LFP	$\hat \beta$		Bias ($\times 1000$)		empSE		ModSE		RMSE		Coverage$^\dagger$	
			$N=300$	$N=1000$	$N=300$	$N=1000$	$ N=300$	$N=1000$	$N=300$	$N=1000$	$N=300$	$N=1000$	$ N=300$	$N=1000$
Cancer	Age	0.553	0.542	0.55	−10.029	−2.559	0.179	0.097	0.177	0.097	0.18	0.097	0.948	0.948
	Sex	0.749	0.8	0.777	50.949	28.345	0.569	0.318	0.573	0.319	0.572	0.319	0.954	0.95
	Year	−0.002	−0.002	−0.003	−0.133	−1.913	0.322	0.17	0.321	0.172	0.322	0.17	0.954	0.956
Other causes	Age	0.49	0.489	0.502	−0.737	12.319	0.045	0.025	0.049	0.028	0.045	0.027	0.968	0.964
	Sex	0.223	0.273	0.235	49.817	12.618	0.134	0.07	0.139	0.077	0.143	0.071	0.934	0.974
	Year	−0.021	0.011	−0.037	31.802	−15.82	0.084	0.047	0.085	0.047	0.09	0.05	0.932	0.92

LFP, least false parameter; empSE, empirical standard error; ModSE, model standard error; RMSE, root mean squared error. $^\dagger$ Acceptable coverage range is calculated as $0.95\pm z_\alpha\sqrt{\frac{0.95\cdot0.05}{500}}=[0.931,0.969]$

## 4. Illustrative example

We illustrated our approach using a data set of 7351 women diagnosed in England with cervical cancer between 2008 and 2010, obtained from the national population-based cancer registry. We limited the sample to those aged between 15 and 89 years, the end of follow-up was set at the 31st of December of 2015 and all individuals had a minimum potential follow-up of 5 years. In this data set, 2255 (30.7%) deaths were observed (all causes considered as the exact COD was not available) while 186 (2.5%) were lost to follow-up. We applied the nonparametric method and the pseudo-observations approach defined in the relative survival setting, and we used the UK life tables stratified by sex, age, calendar year, government office region, and deprivation quintiles.

The covariates of interest for studying their association with the CPr or with the number of LYL due to each cause were: age at diagnosis defined as a continuous variable, and the deprivation quintiles. For the latter, patients were categorized in five socioeconomic groups (from the least deprived group, level 1, to the most deprived group, level 5) using national categories of the income domain of the Index of Multiple Deprivation score (IMD 2004) which is a score defined at the lower super output area level in England.

In this illustration, our aim was to obtain and interpret the regression parameter estimates which quantify the effect of covariates on CPr and LYL due to cancer and other causes. We show how the interpretation changes based on the choice of link function, while we demonstrate in practice the advantages and limitations that were described in Section 2.4.1.

### 4.1. Crude probabilities of death from cancer and other causes

We started with the estimation of the pseudo-observations for CPr from cancer and other causes. Pseudo-observations for each cause (cervical cancer and other causes) were computed at 5 timepoints, which were decided based on the quantiles of the survival time distribution.

We modeled the CPr from cancer and other causes using three different models. All models were simply specified accounting for time-dependent terms (i.e., indicator functions for the four last timepoints at which pseudo-observations were calculated) and two main variables, namely age at diagnosis and deprivation group included as linear terms. Models differed with respect to link functions (cloglog, log, identity) allowing for different interpretations. The working covariance matrix was the same in all models where independence structure was applied. The regression parameter estimates for each model can be seen in Table 3.

**Table 3.** Regression parameter estimates (standard errors) for the direct modeling of the crude probabilities of death from cancer and other causes, as obtained with three models using pseudo-observations with link functions: *cloglog*, *identity,* and *log*, and assuming an independence working covariance structure.

	cloglog		Log		Identity	
	Cancer	Other causes	Cancer	Other causes	Cancer	Other causes
(Intercept)	-2.313(0.072)	-5.835(0.149)	-2.178(0.055)	-5.743(0.14)	0.099(0.01)	0.002(0.001)
*t* = 969 days	0.626(0.028)	0.721(0.018)	0.477(0.023)	0.702(0.017)	0.098(0.004)	0.008(0)
*t* = 1826 days	0.845(0.031)	1.207(0.032)	0.612(0.025)	1.163(0.029)	0.14(0.004)	0.017(0.001)
*t* = 2132 days	0.881(0.032)	1.339(0.036)	0.633(0.026)	1.286(0.033)	0.147(0.004)	0.021(0.001)
*t* = 2487 days	0.927(0.033)	1.474(0.04)	0.659(0.026)	1.41(0.036)	0.156(0.005)	0.025(0.001)
Age$^\dagger$	0.452(0.014)	0.702(0.034)	0.33(0.009)	0.67(0.032)	0.101(0.003)	0.017(0.001)
Deprivation 2	-0.025(0.091)	0.158(0.147)	-0.016(0.067)	0.151(0.141)	-0.001(0.014)	0.002(0.002)
Deprivation 3	0.134(0.087)	0.099(0.137)	0.085(0.063)	0.094(0.131)	0.031(0.014)	0.003(0.002)
Deprivation 4	0.2(0.083)	0.135(0.126)	0.125(0.06)	0.125(0.121)	0.047(0.014)	0.005(0.002)
Deprivation 5	0.186(0.082)	0.223(0.129)	0.12(0.06)	0.203(0.124)	0.043(0.013)	0.008(0.002)

$^\dagger$ (Age at diagnosis-47 (mean age in the data set))/10

In the case of cloglog model, the reported $\hat\beta$ estimates correspond to log subdistribution hazard ratios associated with 1 unit change of a covariate $X$ in the instantaneous rate of the occurrence of an event among those who are event-free or have experienced a competing event (i.e., the subdistribution hazard function). Following the reasoning in Section 2.4.1, we provided only a qualitative description of the results. Age coefficient is positive (0.452), which can be translated to an increase in subdistribution hazard and subsequently, in the probability of dying from cancer with the increase of age. Similarly, the regression parameter for age in the case of other causes was also positive (0.702), indicating an increase in the subdistribution hazard of other causes. Moreover, irrespective of the COD, the most deprived people were associated with a bigger increase in CPr compared to the least deprived, with the only exception being those from deprivation group 2 in the cancer event. Lastly, we can say that for example people from deprivation group 4 who had a larger regression coefficient than those from deprivation 3, had a higher relative change in the incidence of cancer death [see Proof from Ref. (Austin and Fine, 2017)].

Although this interpretation was informative, the model with log link function provided additionally a quantitative interpretation expressed as relative risk. The effect of age was quantified as $\exp(0.33)$, meaning that a 10-year increase in age at diagnosis was associated with an increase in probability of death from cancer by 39% (95% CI 37–42), for a given deprivation group at a given time-point. With respect to other causes, the regression parameter for the effect of a 10-year increase in age indicated an 1.95-fold (95% CI 1.84–2.08) increase in the risk of dying from other causes. Regarding deprivation, by exponentiating the results shown in Table 3, we observed that the most deprived group (deprivation 5) had approximately 1.12 (95% CI 1–1.27) times higher risk of dying from cancer compared to the least deprived group (deprivation 1) at a given time-point after adjusting for age at diagnosis. The corresponding effect on the other causes was 1.23 (95% CI 0.97–1.61).

The identity link model has also the advantage of simple interpretation of the coefficient parameters, providing estimates of risk differences. Therefore, we observed that a 10-year increase in age at diagnosis was associated with an increase in the risk of cancer death (0.101, 95% CI 0.095–0.107) for a given deprivation group at a given time-point [the corresponding estimates for other causes is 0.017 (95% CI 0.015–0.019)]. Furthermore, we observed that for the most deprived group (deprivation 5) the risk difference related to death from cancer was estimated as 0.043 (95% CI 0.02–0.07) compared to the least deprived group (deprivation 1) at a given time-point after adjusting for age. The analogous effect on the other causes was 0.008 (95% CI 0.004–0.012). As we already mentioned in Section 2.4.1, one must be aware of inappropriate predictions when using this model which is true even here, e.g., when trying to predict the probabilities for cancer for someone with an age below 38 years at the 1st time-point.

### 4.2. LYL due to cancer and other causes

The pseudo-observations for LYL from cancer or other causes were estimated within the time period 5 years. A GEE model with identity link function and independence working covariance matrix was applied with age at diagnosis and deprivation group as explanatory variables. According to the model estimates (see Table 4), a 10-year increase in age at diagnosis led to approximately 0.44 (95% CI 0.42–0.47) additional years being lost due to cancer and 0.055 (95% CI 0.051–0.059) due to other causes in the first 5 years. Moreover, people who were more deprived had an increased number of LYL compared to people who were less deprived in the first 5 years, with those in the most deprived group losing around 0.188 (95% CI 0.08–0.3) additional years due to cancer compared to the least deprived.

**Table 4.** Regression parameter estimates (standard errors) for the direct modeling of the number of LYL due to cancer and due to other causes, as obtained with a model for pseudo-observations with *identity* link function and independence working covariance structure.

	Cancer	Other causes
(Intercept)	0.841	0.051
Age$^\dagger$	0.443	0.055
Deprivation 2	0.002	0.005
Deprivation 3	0.144	0.011
Deprivation 4	0.216	0.017
Deprivation 5	0.188	0.025

$^\dagger$ (Age at diagnosis-47 (mean age in the data set))/10

## 5. Discussion

Alternative survival indicators such as CPr and LYL attributed to different causes can prove very useful when communicating survival statistics. That is especially true in the case where the event of interest is cancer whose complexity requires a multi-perspective approach. CPr and LYL are both defined in “real world” and quantify the impact of a covariate on a given event in the presence of other competing events thus, useful to inform about a patient’s prognosis, a treatment choice, or even the development of a control strategy (Charvat *and others*, 2013; Mariotto *and others*, 2014; Pohar Perme *and others*, 2016). The LYL indicator has the additional advantage of being expressed on a time scale, making it easier to communicate the results of analysis to a non-scientific audience (Belot *and others*, 2019). Although these indicators have been well defined and modeled in cause-specific setting, i.e., when the information on COD is available and reliable, a direct modeling of those measures in the relative survival setting was yet unavailable.

In this article, we explored the use of pseudo-observations in modeling these alternative survival measures in relative survival setting with GLMs using the GEE method. This approach enables the user to choose between different link functions and various structures of working covariance matrix.

We evaluated the new approach using simulations and we showed that it performs well for both measures. Regarding CPr, assessment of different models through regression parameters showed good performance regardless of the choice of link function and whilst assuming a simple independence working covariance structure (Klein and Andersen, 2005; Pekár and Brabec, 2018). Regarding LYL, the simulation results displayed good performance for that indicator too, when applying an identity link function and an independence covariance matrix.

The application of the new method to cervical cancer data showed how the covariate effects on the indicators of interest can be derived and interpreted. The models used in the illustration were simple and model misspecification cannot be excluded yet, this study stresses on the interpretation rather than on model building strategies. One interesting further step would be to use goodness of fit tests as recently proposed (Pavlič *and others*, 2018), in order to assess the choice of link function and the functional form of continuous covariates.

In general, this approach offers a useful alternative, especially when considering how the interpretation simplifies when using a model for CPr with a log and identity link function (compared to one with a cloglog function). Although a cloglog link function would give similar interpretations to Fine & Gray model, we advocate the use of log link function with which $\exp(\beta)$ gives an estimate of relative risk (Overgaard *and others*, 2015), and of identity link function which would yield risk differences. This would avoid the pitfalls of interpreting subdistribution hazard ratios (Andersen *and others*, 2012; Austin and Fine, 2017) with the additional advantage of quantitative interpretation of covariate effects on the indicator of interest. However, one must be careful when choosing these link functions as to avoid predictions that go beyond the acceptable range (i.e., [0,1] for probabilities and (0,+$\infty$) for time).

Time-dependent and nonlinear effects can also be easily introduced into the model (Klein and Andersen, 2005). However, inclusion of a time-dependent covariate needs careful consideration, mostly in terms of interpretation due to the fact that the CPr is not a functional of the sole intensity when (nondeterministic) time-dependent covariates are considered (Andersen *and others*, 2003). Knowing the future evolution of such covariates is therefore needed, yet this cannot be practically done when the observed COD is a competing event. Studies that deal with that include a landmarking approach using direct binomial modeling (Grand *and others*, 2018) or a synthesis of separate cause-specific hazard analyses (Beyersmann and Schumacher, 2008) etc., but more research in that direction will be needed in the context of pseudo-observation approach.

There are also other issues in our work which were not explored here, but which could be of possible interest. Firstly, until this point we presented a way to model the pseudo-observations separately for one cause at a time. An alternative choice would be to model them jointly and use a working covariance matrix that reflects the correlation between pseudo-observations of the same cause that would enable the joint estimation of parameters (Andersen, 2013). Secondly, the goal of this article was to show the sensible behavior of the method in practice. This was well confirmed with our simulations, yet more work is needed to derive theoretically the asymptotic properties of the estimators. Thirdly, even though modeling pseudo-observations constitutes a simple and general approach that can simplify survival analysis, it is usually less efficient compared to other methods developed specifically for one indicator of interest. An additional consideration in this approach before applying it to any data, is the assumptions behind relative survival setting (Pavlič and Pohar Perme, 2018), violation of which might result in biased estimators of pseudo-observations and subsequently, an invalid analysis. Lastly, in this study we did not investigate the performance using different covariance matrix structures but we used the independence structure throughout as has been suggested by Klein and Andersen (2005). Impact of other structures on the results would be an interesting further methodological development.

In summary, our approach based on pseudo-observations in relative survival setting demonstrated nice frequentist properties on estimating the crude probabilities of death and the LYL from different causes in realistic situations. These two indicators along with other frequently reported measures like net survival can improve the understanding of the nature and mechanism of competing events. Their computation in relative survival setting is quite important as routinely collected population-based data often suffer from unreliable or unavailable information of the COD. The advantage of the pseudo-observation approach to provide covariate effects directly affecting the indicators of interest in the relative survival setting, makes the method appealing to the user. However, one should be aware that this approach might be prone to a longer computational time (especially in the case of big data sets) compared to conventional methods. A guide that provides the code for applying the method in R-software is also provided.

## Supplementary Material

kxaa017_Supplementary_Data

## Software

The implementation R-code simulation code, and sample data are available at https://github.com/pseudorel/supp_material.git.
