# Supplementary material for: Direct modeling of the crude probability of cancer death and the number of life years lost due to cancer without the need of cause of death: a pseudo-observation approach in the relative survival setting
Source: Biostatistics. 2020 May 6;23(1):101–19. doi: 10.1093/biostatistics/kxaa017 (PMC8759449; doi:10.1093/biostatistics/kxaa017)
Supplement: kxaa017_Supplementary_Data [file kxaa017_supplementary_data.pdf]

Biostatistics (2020), **0**, 0, *pp.* 1–4
doi:10.1093/biostatistics/Supplementary˙material

Supplementary material A to "Direct modelling of the crude probability of cancer death and the number of life-years lost due to cancer without needing the cause of death: a pseudo-observation approach in the relative survival setting"

DIMITRA-KLEIO KIPOUROU[a*],
MAJA POHAR PERME[b], BERNARD RACHET[a], AURELIEN BELOT[a]
[a] *Cancer Survival Group, Faculty of Epidemiology and Population Health, Department of Non-Communicable Disease Epidemiology, London School of Hygiene & Tropical Medicine, London, WC1E 7HT, UK*
[b] *Institute for Biostatistics and Medical Informatics, Faculty of Medicine, University of Ljubljana, Ljubljana, Slovenia*

1. Simulating data using subdistribution hazard and life tables

Haller and Ulm (2014) showed how to use pre-specified a subdistribution hazard (SDH) combined with a cause-specific hazard (CSH) in order to simulate competing risks data in cause-specific setting. Here, we show how we could adapt this approach to the relative survival setting in order to use a SDH and life tables in order to describe cancer and the other causes, respectively.

The simulation algorithm has six steps:

1. Generate a certain population of individuals with desirable characteristics. These should include all covariates of interest ($\boldsymbol{X}$) in addition to those used to match the life table with population ($\mathbf{z}$)

2. Obtain the expected mortality λ_P from life tables which are stratified by $\mathbf{z}$

3. Estimate the cancer-specific hazard λ_1 through subdistribution hazard γ_0 and λ_P

4. Derive individual survival times

5. Apply censoring (administrative/drop outs)

6. Determine final survival times

© The Author 2020. Published by Oxford University Press. All rights reserved. For permissions, please e-mail: journals.permissions@oup.com

Generate individual covariates

We start by generating a cohort with pre-defined characteristics such as demographic or other information regarding treatment, disease stage, etc. These variables must include all covariates of interest ($\boldsymbol{X}$) in addition to those used to match the population with the life table, ($\mathbf{z}$).

We may generate our own population with characteristics based on pre-defined distributions or using an existing dataset based on real data. We do not need to use many populations; by using the algorithm described below, the survival time and the event types for each individual will differ from dataset to dataset despite individuals being exactly the same in each dataset.

Obtain expected mortality, λ_P

λ_P is usually obtained from life tables built by national statistics institutes and stratified on some sociodemographic variables (such as age, sex, calendar year, deprivation and region). Expected mortalities are changing annually hence, we assume that λ_P follows a piecewise exponential distribution.

Estimate excess-hazard, λ_1

We start by setting a model on SDH for the cause of interest (here denoted as γ_0). A wide range of models can be used to specify γ_0, from simple to more advanced parametric models.

The estimation of λ_1 can be later achieved based on γ_0 and λ_P after adapting equation (9) found in Haller and Ulm (2004) after assuming that $\lambda_P := \lambda_2$. Thus, when $\lambda_P(t|\mathbf{z})$ and $\gamma_0(t|\boldsymbol{X})$ are specified (with $\mathbf{z}_i \subset \boldsymbol{X}_i$), then $\lambda_1(t|\boldsymbol{X})$ may be obtained via

$$\lambda_1(t|\boldsymbol{X}_i) = \frac{\gamma_0(t|\boldsymbol{X}_i)\exp(-\Gamma_0(t|\boldsymbol{X}_i) + \Lambda_P(t|\mathbf{z}_i))}{1 - \int_0^t \gamma_0(u|\boldsymbol{X}_i)\exp(-\Gamma_0(u|\boldsymbol{X}_i) + \Lambda_P(u|\mathbf{z}_i))\mathrm{d}u} \tag{1.1}$$

For this expression to hold, additional constraints should be satisfied (please see Section 3.2 from Haller and Ulm (2004)).

Generate survival times, T

We choose to generate the individual survival times using the inversion method as shown below.

Let us suppose that $\Lambda(t) = \int_0^t \sum_{j=1}^J \lambda_j(u)\mathrm{d}u$ is the cumulative all-cause hazard, which is an increasing and invertible function as is the distribution of survival time T. Without distinguishing between causes, it holds that

$$F(t) = P(T \leqslant t) = 1 - \exp(-\Lambda(t))$$

If F^{-1} is the inverse of F and $F(t)$ is uniformly distributed on $[0, 1]$ and then,

$$P(F(T) \leqslant u) = P(T \leqslant F^{-1}(u)) = F(F^{-1}(u)) = u, \quad u \in [0, 1]$$

Assuming a random variable U with a uniform distribution on $[0, 1]$, then $F^{-1}(U)$ has the same distribution as T. Thus, all we need to do to generate survival times is to compute the $F^{-1}(U) = \Lambda^{-1}(\ln(1 - U))$. If we cannot find the $\Lambda^{-1}(t)$, then numerical inversion may be an alternative (please see more details in Beyersmann et. al (2011)).

Generate event types, ϵ

Using a Bernoulli experiment we determine the event type of each individual with probabilities $\frac{\lambda_1(t|\boldsymbol{X})}{(\lambda_1(t|\boldsymbol{X})+\lambda_P(t|\mathbf{z}))}$ for a cancer event and $\frac{\lambda_P(t|\mathbf{z})}{(\lambda_1(t|\boldsymbol{X})+\lambda_P(t|\mathbf{z}))}$ for other causes.

Apply a censoring mechanism, C

The censoring mechanism may be a combination of administrative censoring and a random drop-out mechanism.

Determine final survival times and event types

The final survival times (T_S) will be given as the $\min(T; C)$. If the final survival time is equal to the censoring time, then ϵ takes the value 0, otherwise it remains as it is.

Sample code

Sample code for the simulation described above can be found in `https://github.com/pseudorel/supp_material`.

2. Estimation of least false parameters

Below we show how we estimated the LFP. The formulae described below were applied to the large dataset (please see more details in Section 3.2) where we do not consider any drop outs. With this proof we want to show that even if the calculations start from the leave-one-out estimator, what is actually estimated is *independent* of the pseudo-observations.

In case of no censoring

$$
\begin{aligned}
\hat{F}_C(t) &= \int_0^t \hat{S}_O(u-)\mathrm{d}\Lambda_E(u) \\
&= \int_0^t \frac{1}{n}\sum_{k=1}^n I(T_k \geqslant u)\frac{\sum_{k=1}^n \mathrm{d}N_k(u) - \sum_{k=1}^n Y_k(u)\mathrm{d}\Lambda_P(u,\mathbf{z}_k)}{Y(u)} \\
&= \frac{1}{n}\int_0^t \sum_{k=1}^n I(T_k \geqslant u)\frac{\sum_{k=1}^n \mathrm{d}N_k(u) - \sum_{k=1}^n Y_k(u)\mathrm{d}\Lambda_P(u,\mathbf{z}_k)}{\sum_{k=1}^n I(T_k \geqslant u)} \\
&= \frac{1}{n}\int_0^t \sum_{k=1}^n \mathrm{d}N_k(u) - \sum_{k=1}^n Y_k(u)\mathrm{d}\Lambda_P(u,\mathbf{z}_k) \\
&= \frac{1}{n}\sum_{k=1}^n \int_0^t \mathrm{d}N_k(u) - \int_0^t Y_k(u)\mathrm{d}\Lambda_P(u,\mathbf{z}_k) \\
&= \frac{1}{n}\sum_{k=1}^n \left(N_k(t) - \int_0^t Y_k(u)\mathrm{d}\Lambda_P(u,\mathbf{z}_k)\right)
\end{aligned}
\tag{2.2}
$$

Applying that to the leave-one-out estimator

$$
\begin{aligned}
\tilde{F}_{C,i}(t) &= n \cdot \hat{F}_C(t) - (n-1) \cdot \hat{F}_C^{(-i)} \\
&= n \cdot \left(\frac{1}{n} \sum_{k=1}^{n} \left(N_k(t) - \int_0^t Y_k(u) \mathrm{d}\Lambda_P(u, \mathbf{z}_k) \right) \right) - (n-1) \cdot \left(\frac{1}{n-1} \sum_{\substack{k=1 \\ k \neq i}}^{n} \left(N_k(t) - \int_0^t Y_k(u) \mathrm{d}\Lambda_P(u, \mathbf{z}_k) \right) \right) \\
&= N_i(t) - \int_0^t Y_i(u) \mathrm{d}\Lambda_P(u, \mathbf{z}_i)
\end{aligned} \tag{2.3}
$$

Similarly for the other causes

$$
\begin{aligned}
\hat{F}_{P,i}(t) &= \int_0^t \hat{S}_O(u-) \mathrm{d}\Lambda_P(u) \\
&= \int_0^t \frac{1}{n} \sum_{k=1}^{n} I(T_k \geqslant u) \frac{\sum_{k=1}^{n} Y_k(u) \mathrm{d}\Lambda_P(u, \mathbf{z}_k)}{Y(u)} \\
&= \frac{1}{n} \int_0^t \frac{\sum_{k=1}^{n} Y_k(u) \mathrm{d}\Lambda_P(u, \mathbf{z}_k)}{Y(u)}
\end{aligned} \tag{2.4}
$$

Hence, the pseudo-observation for the other CPr related to other causes is estimated as

$$
\begin{aligned}
\tilde{F}_{P,i}(t) &= n \cdot \hat{F}_P(t) - (n-1) \cdot \hat{F}_P^{(-i)} \\
&= n \cdot \left(\frac{1}{n} \sum_{k=1}^{n} \left(\int_0^t Y_k(u) \mathrm{d}\Lambda_P(u, \mathbf{z}_k) \right) \right) - (n-1) \cdot \left(\frac{1}{n-1} \sum_{\substack{k=1 \\ k \neq i}}^{n} \left(\int_0^t Y_k(u) \mathrm{d}\Lambda_P(u, \mathbf{z}_k) \right) \right) \\
&= \int_0^t Y_i(u) \mathrm{d}\Lambda_P(u, \mathbf{z}_i)
\end{aligned} \tag{2.5}
$$
